# Social Inequalities in the Association between Social Infrastructure and Mental Health: An Observational Cross-Sectional Analysis of Children and Adolescents in Germany

**DOI:** 10.3390/ijerph19116760

**Published:** 2022-06-01

**Authors:** Katharina Stahlmann, Emily Mena, Ronny Kuhnert, André Conrad, Gabriele Bolte

**Affiliations:** 1 Department of Medical Biometry and Epidemiology, University Medical Center Hamburg-Eppendorf, 20251 Hamburg, Germany; k.stahlmann@uke.de; 2 Department of Social Epidemiology, Institute of Public Health and Nursing Research, University of Bremen, 28359 Bremen, Germany; gabriele.bolte@uni-bremen.de; 3 Health Sciences Bremen, University of Bremen, 28359 Bremen, Germany; 4 Department of Epidemiology and Health Monitoring, Robert Koch-Institute, 13353 Berlin, Germany; kuhnertr@rki.de; 5 German Environment Agency (Umweltbundesamt), 14195 Berlin, Germany; andre.conrad@uba.de

**Keywords:** mental health, built environment, child, adolescent, social inequality

## Abstract

The mental health (MH) of especially children and adolescents with low socioeconomic status (SES) benefits from access to greenspaces. This study aimed at investigating social inequalities in the association between several types of social infrastructure (SI) and MH in children and adolescents. The sample comprised 12,624 children and adolescents of the KiGGS Wave 2 study (2014–2017). KiGGS provided information on SI (access to playgrounds, sports fields, swimming pools, parks) for all children and the environmental module (GerES V) within KiGGS on the walking time to SI for a subsample. Social inequality was measured by parental SES and the German Index of Socioeconomic deprivation and MH by the Strengths and Difficulties Questionnaire. Ordinal logistic regression analyses showed that access to fewer SI places was associated with higher odds of MH problems. Children and adolescents experiencing high (but not medium or low) socioeconomic deprivation at the municipal level were more likely to have MH problems when having less access to SI places. At the individual level, MH problems in high- and low-SES, but not medium-SES children and adolescents were associated with no access to SI places. Children and adolescents from high socioeconomically deprived areas and with low and high SES might benefit from high-availability SI.

## 1. Introduction

Mental health problems in childhood and adolescence are associated with not only more pronounced health damaging behavior [1], but also mental [1] and physical [2] burden in early and later adulthood. According to Kessler et al. [3], half of all lifetime mental health problems start in childhood. Many different factors on individual, family, and neighborhood levels affect children’s mental health. For instance, the experience of stressful life events [4] or parental mental health problems [5] pose a risk for mental health difficulties, while coping strategies and family routines have a beneficial impact on mental health [5]. In particular, children with low socioeconomic status (SES) are more likely to be exposed to detrimental risk factors [4,5] and to face more barriers in the access to mental healthcare [6] than those with high SES. In addition, lack of material resources is related to an early onset of mental health problems, and parental education is related to severity and persistence [6]. On a neighborhood level, previous research observed a higher prevalence of mental health problems in children living in socioeconomically deprived neighborhoods [7,8]—especially urban ones [9]—which may be due to lower social cohesion [10] and worse physical conditions [8,11] in these areas. Worldwide, the prevalence of mental disorders in children and adolescents was estimated to be 11.3–15.9% in 2015 [12]. Currently, the COVID-19 pandemic has led to a strong increase in childhood mental health problems in Germany [13]. In particular, socioeconomically disadvantaged children and adolescents, who already show a high prevalence [14], are at a further increased risk of mental health problems [13]. Consequently, research and prevention policies should focus on the mental health of disadvantaged children and adolescents [14].

While the influence of social determinants has been extensively studied [15], research regarding the effect of built environment, commonly defined as the physical environment designed by humans [15,16], on mental health is scarce. Changing the neighborhood’s built environment may help to reduce health inequality and promote health equity by targeting especially those who would benefit most from these changes and is, thus, an important public health measure [17]. While several systematic reviews have inferred a positive effect of public and private greenspace on mental health in children and adolescents [16,18,19], studies looking at further aspects of local social infrastructure (SI) are scarce [16]. Local services such as childcare, educational, cultural, and sport services—particularly a good mix of them—are essential for adult wellbeing [20] because of their promotion of social interactions [15,20]. The few studies investigating the effect of youth specific SI on children’s mental health reported mixed results. Whereas a higher distance to child-specific services was associated with better child mental health on community level in an ecological study in Australia [21], Butler et al. [8] observed that access to one and three (but not zero or two) amenities (sidewalks, parks or playgrounds, recreation/community centers, and libraries) is related to a higher likelihood of anxiety/depression in US children and adolescents compared to access to four amenities. These inconclusive findings about the influence of youth-specific SI might be due to the fact that the effect differs by population subgroup. For instance, compared to high-SES children, mental health of low-SES children and adolescents from Lithuania, Scotland, England, and the USA benefited more from proximity to parks [22], private garden access [23], and access to greenspace [24,25], respectively. In Germany, low-SES children and adolescents are two times more likely than high-SES ones to walk more than 10 min to the nearest greenspace [26] and to live in disadvantaged neighborhoods [11], with both components of social inequality contributing to worse mental health outcomes [11]. Moreover, socioeconomically disadvantaged neighborhoods have often fewer and lower-quality services [27] and provide less access to greenspace [28] than advantaged ones. Apart from SES, some differences by sex may also prevail. While boys’ peer problems and total difficulties were negatively associated with access to parks and private garden in Scottish children aged 4 to 6, access to total natural space and garden was associated with less hyperactivity, total difficulties, peer problems (only total natural space), and more prosocial behavior (only total natural space) in girls [23]. While rural areas provide much open natural space, urban areas are more densely built up and lack greenspaces apart from parks [29] which may contribute to differences in the effect of SI on mental health between rural and urban children [30].

This underscores the importance of looking at socioeconomic factors when analyzing the health relevance of built environment.

Therefore, this cross-sectional study aims at investigating social inequalities in the association between youth-specific SI and children’s and adolescents’ mental health. In addition to examining how SI affects mental health, this study identifies for which SES group SI matters the most. It is hypothesized that low-SES children and adolescents, as well as those living in high socioeconomically deprived municipalities, are more likely to have mental health difficulties than those with high SES and those from low socioeconomically deprived municipalities when having less access to youth-specific SI.

## 2. Materials and Methods

### 2.1. Design and Procedure

We conducted an exploratory cross-sectional study using multiple data sources. Core individual data stemmed from the second wave of the German Health Interview and Examination Survey for Children and Adolescents (KiGGS). As part of the health monitoring conducted by the Robert Koch Institute (RKI), the KiGGS wave 2 study provides nationwide representative cross-sectional data of 15,023 children and adolescents aged 0–17 years with permanent residence in Germany. They were recruited by a two-stage stratified random sampling between 2014 and 2017 (response rate was 40.1%) [31]. The ethics committee of the Medical University Hannover (Medizinische Hochschule Hannover) approved the KiGGS wave 2 study (No. 2275-2014). Participation was voluntary, and all children, adolescents, and their parents were informed about the aim, scope, and data protection procedure of the study before giving their informed consent [32]. More detailed methodological information can be found in Mauz et al. [32] and Hoffmann et al. [31].

Neighborhood social inequality was described by version 1.0 of the German Index of Socioeconomic Deprivation (GISD) developed by the RKI [33] on the association of municipalities level (Gemeindeverbundebene). This index is freely available on GESIS [34]. GISD data were linked to the individual KiGGS data by municipality.

In addition, Indicators and Maps for Spatial and Urban Development (INKAR) data [35] provided more information on the characteristics of the municipalities. INKAR is an interactive online database operated by Germany’s Federal Institute for Research on Building, Urban Affairs, and Spatial Development. It contains regional statistics for Germany and Europe on different regional levels and provides information on various domains, such as transport, demographics, land use, social services, and medical care [36].

Lastly, individual data of 2294 children and adolescents of the fifth wave of the German Environmental Survey of Children and Adolescents (GerES V, 2014–2017), the environmental module of the KiGGS wave 2, were included. GerES V is part of the German Federal Health-Related Environmental Monitoring and provides data on the exposure to environmental stressors and other health-relevant aspects. The data protection policy was approved by the Federal Commissioner for Data Protection and Freedom of Information. The GerES V study was further approved by the ethics committee of the Medical Association (Ärztekammer) Berlin (No. Eth-14/14). Participation was voluntary, and all children, adolescents and their parents were informed about the aim, scope, and data protection procedures of the study before giving their informed consent [37]. More detailed information regarding GerES V can be found in Schulz et al. [37].

### 2.2. Social Infrastructure

The availability of youth-specific SI was examined as an exposure variable and measured in the KiGGS study by the question “Are there play or sports facilities in your current location that are within easy reach of your child?”, which was answered by a parent or legal guardian in a self-administered questionnaire. Three answer options (“yes”, “no”, and “I do not know”) were available for each item (playground, sports field/hall, swimming pool/hall, and park). The answers “I do not know” (*n* = 63 for access to playgrounds, *n* = 124 for sport fields, *n* = 78 for swimming pools, and *n* = 50 for parks (of all children and adolescents aged 3 to 17 with data on the Strength and Difficulties Questionnaire (SDQ))) were recoded as “no” since it was assumed that if the respondent did not know about the respective place, it is not in the immediate living environment and was not visited by the child/adolescent. Similar to a common procedure applied to food frequencies questionnaires [38], missing values were coded as “no” if the respondent checked only “yes” or left the answer box blank (missing) for one or more other items. To capture the mix of social infrastructure [20], a score (KiGGS SI score) was calculated by summing the response values (yes = 1, no = 0) of each item, resulting in a score with values from 0 (no SI places available) to 4 (all four SI places available).

More detailed information on the distance to SI was obtained in GerES V. Parents or legal guardians were asked in a computer-assisted interview “How long does it take [your child] to reach the following places/facilities by foot?” For each item (playground, sports field/hall, indoor playground, swimming pool/hall, park/public greenspace, forest, beach/lake/stream/river (in the following blue spaces), and bus or train stop), answer categories were 1–5 min, 6–10 min, 11–20 min, 21–30 min, more than 30 min, and I do not know. Again, a score (GerES V SI score) was created by dichotomizing the responses according to considerations by Rehling et al. [26] with respect to public greenspace into 10 min (deemed as “easy walking distance” by the European Commission [39]) or less (1) and more than 10 min (0) and summing the values of each item. The resulting score gives the number of available close (≤10 min) SI places and ranged from 0–8.

### 2.3. Social Inequality

Social inequality on the municipal level was measured by the GISD [33]. Using data from the INKAR database, the GISD aggregates eight educational, occupational, and income-related indicators and is available for several regional levels, as well as for different years. The index is provided as a continuous score or categorized into quintiles or tertiles (based on quintiles), with the latter being used for this study (low = first quintile, middle = second to fourth Quintile, and high = fifth quintile) [40]. The most recent data on the smallest regional level (associations of municipalities) from 2012 were selected. More detailed information on the construction of this index can be found in Kroll et al. [33,40].

On the individual level, social inequality was captured by parental socioeconomic status provided in the KiGGS wave 2 dataset. The composite multidimensional index was derived on the basis of parental information on education, occupational status, and income [41]. Index data, categorized into tertiles (low = first quintile, middle = second to fourth quintile, and high = fifth quintile), were used for the main analyses and data of the single continuous indicators for a sensitivity analysis. With reference to Weinberg et al. [42] who observed an increasing importance of adolescents’ (aged ≥ 11 years and <18 years) subjective socioeconomic status (SSS) with respect of mental health, the self-reported SSS was included in an adolescents’ subgroup analysis instead of the parental SES. A detailed explanation of the methodology of the construction of the SES index and the SSS in the KiGGS study can be found in Lambert et al. [41].

### 2.4. Mental Health

Data on the SDQ by Goodman [43], which was included in the KiGGS study, were used to analyze children’s and adolescents’ mental health. It is a brief and valid screening questionnaire (Cronbach’s alpha = 0.82 for total difficulties [44]; confirmatory factor analysis showed a CFI of 0.925 and 0.918, a TLI of 0.915 and 0.907, and an RMSEA of 0.049 and 0.047 for boy and girls, respectively [45]) including 25 items out of the five subscales measuring emotional symptoms, conduct problems, peer problems, inattention/hyperactivity, and prosocial behavior. A total difficulties score was calculated on the basis of the values obtained in the first four subscales (excluding prosocial behavior) [43]. According to international reference values [46], the total difficulties and the subscales score were categorized into normal (a total difficulties score of 0–13), borderline (a total difficulties score of 14–16), and abnormal (a total difficulties score of 17–40). This study used the SDQ data of the self-administered questionnaire completed by the child’s/adolescent’s parent. The SDQ was used because of its validation within the KiGGS baseline sample [45] and its widespread application for child and adolescent mental health in current studies of environmental exposures [22,23,24]. Its utilization is recommended for comparability with other studies [19].

### 2.5. Covariates

On the basis of current literature, the following covariates were selected from the KiGGS dataset: sex (binary: male/female), age (in years), migration background (none, one-sided, two-sided), parental marital status (married and cohabitating, married and separate living, not married, divorced, and widowed). In addition, the municipality’s spatial location (measured on a municipality level: very central, central, peripheral, and very peripheral) was extracted from the INKAR database. For one of the sensitivity analyses, these covariates were extended by the variable “living in the municipality since birth” (yes/no; provided by the KiGGS study).

### 2.6. Statistical Analysis

First, descriptive statistics for sociodemographic, SI, and mental health variables were obtained for the full and sex-stratified sample. Results of multilevel analyses with “proc glimmix” indicated that there is not enough variation in the outcome that could be attributed to the municipality level for some mental health subscales and a high maximum gradient. Therefore, ORs with their 95% CIs were calculated by conducting a multiple ordinal logistic regression with “proc surveylogistic” in SAS instead. This procedure aims at complex survey data (as the KiGGS study) and controls for the clustering in municipalities. In advance, the proportional odds assumption and multicollinearity were tested. In a baseline model, the association between SI and mental health, adjusted for the GISD, individual SES, and the covariates, was estimated. Next, interaction terms between SI with GISD and with individual SES, as well as with sex and age were included as previous studies indicated differences in mental health between sex and age groups [23,47]. Stratified analyses were performed if the *p*-value of the interaction term was ≤0.1. Thus, stratified analyses by GISD, individual SES, and age (<11 years vs. ≥11 years) were conducted.

For sensitivity analyses, the covariate set was augmented with the variable “living in the municipality since birth” which was not included into the baseline model due to a high number of missing values. Because of the potential multicollinearity between spatial location and urbanicity (Spearman’s correlation coefficient s = 0.6) and a moderate correlation between the SI score and urbanicity (s = 0.26), urbanicity was not included as a covariate but was stratified by (metropolitan/medium city (denoted as urban) vs. (larger) small city/rural area (denoted as rural)) in another sensitivity analysis. Next, the single SES indicators (occupational, educational, and income-related subscale) were included into the baseline model instead of the composite SES index as recent studies have stated that each dimension is slightly differently associated with mental health [4,6,7]. Despite available weighting factors for the KiGGS study, unweighted regressions were conducted as they yield correct standard errors and are more efficient [48]. Although “proc surveylogistic” was opted for in the main analyses, a multilevel analysis using “proc glimmix” was performed as sensitivity analysis. According to the recommendation of Moineddin et al. [49], the sample was, for this purpose, restricted to children and adolescents living in municipalities with at least 50 observations to obtain unbiased fixed effect parameters. In addition to the single-score items, all analyses examined the KiGGS SI score as categorical variables since a previous study indicated a nonlinear relationship [8]. In another sensitivity analysis, the score was included as continuous variable instead to prove the linear trend found in the main analyses.

For the exploratory subgroup analysis with GerES V data, the association between the GerES V SI score, as well as its individual items, and mental health outcomes was examined in separate analyses with the same covariates set as the main analyses. Owing to the smaller sample size in GerES V, a binary logistic regression—combining the categories borderline and abnormal—was conducted.

The analyses of the full sample investigated the total difficulties score and all single mental health subscales as dependent variable since previous studies observed differing associations between built environment and children’s mental health across these single subscales [19,22,23]. Thus, our results can be better compared to existing research. We used a complete case dataset for all analyses except for some sensitivity analyses (namely the multilevel analysis and the analyses adjusted for “living in the municipality since birth” and SSS, respectively). Statistical analyses were conducted with SAS 9.4 (SAS Institute, Cary, NC, USA).

## 3. Results

### 3.1. Demographic Characteristics

The final sample comprised 12,624 children and adolescents (49.6% male, mean age 10.1 ± 4.2) from 164 municipalities (mean observations per cluster 77.0 ± 33.5; 27 clusters (with 1148 children and adolescents in total) having <50 observations). Most children and adolescents had married and cohabitating parents (76.9%), had no migration background (80.7%), and lived in central and in urban areas. More than half of the sample had a medium SES (61.8%), while one-quarter had a high SES. A similar distribution can be observed for the municipality’s socioeconomic deprivation (GISD). There were no sex differences in demographic characteristics (Table 1).

### 3.2. Distribution of Social Infrastructure and Mental Health Outcomes

On average, children and adolescents had access to 2.9 (±1.1) of the four (playground, sports field, swimming pool, and park) KiGGS SI places. Except for swimming pools, which half of the sample had access to, all other KiGGS SI places were available for the majority of children and adolescents. Most children had normal mental health in terms of total difficulties (88.1%). Overall, a slightly higher proportion of girls showed normal mental health compared to boys (89.6% vs. 86.4%). Concerning the different subscales, boys had a higher prevalence of combined borderline and abnormal behavioral problems, hyperactivity/inattention, peer problems, and prosocial behavior compared to girls, while girls had a higher prevalence of combined borderline and abnormal emotional symptoms than boys (Table 1).

### 3.3. The Association between Social Infrastructure and Mental Health

A gradient in the association of the KiGGS SI score with total difficulties and peer problems could be observed in the full sample (ORs above 1 indicate higher odds of having borderline or abnormal mental health problems). For instance, children and adolescents with access to three, two, one, and zero KiGGS SI places were 14% (OR 1.14, 95% CI: 0.99, 1.33), 37% (OR 1.37, 95% CI: 1.17, 1.61), 36% (OR 1.36, 95% CI: 1.13, 1.65), and 59% (OR 1.59, 95% CI: 1.19, 2.11), respectively, more likely to have worse total difficulties compared to those with access to all four places (Table 2). Regarding emotional symptoms and hyperactivity/inattention, access to fewer than four SI places was associated with higher odds of mental health problems, but no clear gradient could be noted. However, a linear relationship between the KiGGS SI score and all mental health domains (except for behavioral problems) was identified in the analysis of the KiGGS SI score as a continuous variable (Table S1). Looking at the KiGGS SI places separately, higher odds of worse total difficulties could be found for having no access to sports fields, swimming pools, and parks. These KiGGS SI places were also associated with some of the single mental health domains apart from prosocial behavior (Table 2).

### 3.4. Social Inequalities in this Association

**Figure 1:**
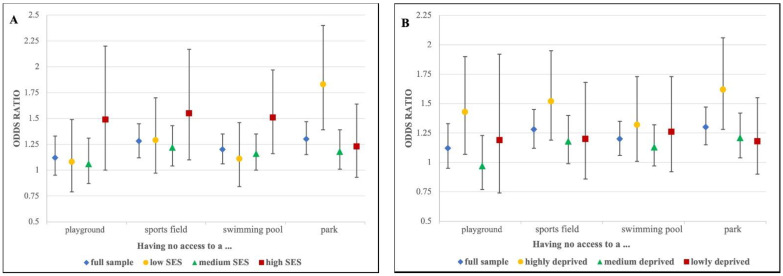
Odds ratios and 95% confidence intervals for total difficulties in children and adolescents who have no access to selected social infrastructure places compared to those with access: (**A**) stratification by socioeconomic status (SES); (**B**) stratification by municipal socioeconomic deprivation.

Corresponding to interaction terms in the multivariable analysis between SI variables and SES (for KiGGS SI score *p* = 0.08 and park access *p* = 0.02), differences in the association between social infrastructure and mental health were observed across SES strata (Table 3 and Tables S2–S4). The gradient in the association between the KiGGS SI score and total difficulties was most pronounced in children and adolescents with high SES (OR 1.65 (95% CI: 1.23, 2.23), OR 1.81 (95% CI: 1.29, 2.55), OR 1.79 (95% CI: 1.00, 3.18), and OR 2.07 (95% CI: 1.01, 4.23) for access to three, two, one, and zero KiGGS SI places, respectively) and least pronounced in those with medium SES. Compared to those with access to all four SI places, low-SES children and adolescents had slightly lower odds ratios than high SES ones for mental health problems when having access to three, two, one, and zero places (OR 1.21 (95% CI: 0.85, 1.73), OR 1.73 (95% CI: 1.22, 2.44), OR 1.52 (95% CI: 1.00, 2.31), and OR 1.71 (95% CI: 0.95, 3.07), respectively). While SI was foremost associated with emotional symptoms and, to a smaller extent, with prosocial behavior in low-SES children and adolescents, it was more closely related to behavioral problems, total difficulties, and, to a certain degree, emotional symptoms in high-SES ones (Tables S2–S4). While medium- and high-SES children and adolescents showed higher odds of worse mental health when lacking access to sports fields and, in the case of high-SES ones, also swimming pools and playgrounds, the mental health of those with low SES was associated with access to parks (Figure 1A).

In addition to the interaction between SI and SES, an interaction between SI and the GISD was detected (for KiGGS SI score *p* = 0.06 and playground access *p* = 0.08). Compared to the analyses stratified by individual socioeconomic status (SES), stratification by socioeconomic deprivation of the municipality (GISD) revealed slightly different results. The strongest gradient of the KiGGS SI score could be observed for all mental health domains in children and adolescents living in municipalities with high socioeconomic deprivation (regarding total difficulties: OR 1.52 (95% CI: 1.08, 2.13), OR 1.75 (95% CI: 1.17, 2.62), OR 1.71 (95% CI: 1.15, 2.55), and OR 2.86 (95% CI: 1.74, 4.70) for access to three, two, one, and zero KiGGS SI places). While those living in medium deprived municipalities had moderately elevated ORs for some KiGGS SI score categories, children and adolescents from low socioeconomically deprived municipalities did not show elevated ORs (Table 3). Similar to stratification by individual SES, children and adolescents living in high socioeconomically deprived areas—and, to a smaller extent, those from medium deprived areas—were more likely to have worse mental health when having no access to parks and, to a lesser extent, when lacking access to sports fields and playgrounds (Table 3 and Tables S5–S7, Figure 1B).

### 3.5. Differences by Age and Urbanicity

An interaction between age and swimming pool access (*p* = 0.01), but not between age and other SI variables or between sex and SI variables could be observed. ORs for the association of the KiGGS SI score and the single KiGGS SI places with total difficulties were higher in children (<11 years) and in urban children and adolescents compared to adolescents (≥11 years) (Table S8) and those living in rural areas (Table S9), respectively.

### 3.6. Sensitivity Analyses

The sensitivity analyses additionally adjusted for the variable “living in the municipality since birth” (Table S10), for the single SES indicators (Table S10) and for the SSS within the adolescents’ subgroup (Table S8), revealed effect estimates close to those of the main analysis. The multilevel regression analysis showed generally similar ORs, a few of which were slightly higher for the KiGGS SI score and its single variables than in the main analysis (Table S10).

### 3.7. The Explorative Analysis of the GerES V Sample

The GerES V sample comprised 2106 children and adolescents (49.5% male, mean age 10.1 ± 4.1) in which demographic and socioeconomic characteristics were similar distributed as within the full KiGGS sample (Table S11). Despite nonsignificant effect estimates for the GerES V SI score, a gradient of higher ORs for worse mental health difficulties for access to fewer GerES V SI places within 10 min walking distance could be noted for total difficulties, emotional symptoms, and hyperactivity/inattention (Table S12). For total difficulties and emotional symptoms, the continuous GerES V SI score affirmed this trend (Table S1). Looking at the single GerES V SI places, a linear trend of higher odds of mental health difficulties with increasing walking time could be found for the association between blue spaces and total difficulties, as well as between emotional symptoms and public greenspace, forest, public transport station, and blue spaces, respectively (Tables S1 and S12).

## 4. Discussion

This study was aimed at estimating social inequalities in the association between youth specific SI and mental health in children and adolescents and to detect subgroups whose mental health is more affected by SI. Overall, this study showed that the odds of having worse mental health difficulties increase with access to fewer KiGGS SI places, especially with respect to total difficulties and peer problems. This association is mostly driven by having no access to parks or sports fields. However, stratified analyses indicated considerable differences between several socioeconomic subgroups.

The association between SI and mental health was strongest in high- and low-SES children and adolescents, as well as in those living in high socioeconomically deprived areas. The association was weaker in medium SES children and adolescents, as well as in those living in low and medium deprived areas.

The analysis of the smaller GerES V subsample hinted also at a gradient of higher odds of total difficulties, emotional symptoms, and hyperactivity/inattention for access to fewer SI places within 10 min walking time. In addition, a higher reported walking time to parks, forests, and blue spaces was associated with more mental health problems.

As the mental health of children and adolescents living in high socioeconomically deprived areas is most strongly associated with access to SI and, thus, they might benefit most of access to these places, the second part of the hypothesis was confirmed. With respect to individual SES, it could be observed that the mental health of both low-SES and high-SES children is related to SI. Hence, the first part of the hypothesis could not be affirmed.

The negative association between park access and mental health difficulties is in line with recent systematic reviews about the effect of public greenspace on children’s and adolescents’ mental health [16,18,19]. Furthermore, results of the GerES V sample confirmed other systematic reviews in the positive association of the distance to greenspaces [19] and blue spaces [50] with mental health difficulties. Compared to playgrounds, open natural spaces, such as forests, blue spaces, and parks, favor more creative, adventurous, social, and challenging play, as well as mental recreation [51], which may explain the small effects for playground access seen in this study. Moreover, this study’s results contradict the findings of Christian et al. [21], who observed a lower vulnerability in Australian children (on community level) with higher distance to other types of child-specific places (child-center-based care, school grounds, and parks) in an ecological study. However, their OR estimates were close to 1. The discrepancy between Christian et al.’s [21] study and this study may be attributed to the different study design, another operationalization of children’s mental health, their focus on only children in their first year of school, and partly different SI places. Additionally, Germany and Australia differ substantially regarding the topography surrounding the study’s location (Perth in Australia vs. 167 municipalities across Germany) which might also influence the association between SI and mental health. Furthermore, the KiGGS SI and GerES V SI places are not only independently associated with mental health, but also cumulatively result in a linear trend of higher odds for mental health difficulties with fewer available SI places. This was also observed for wellbeing of New Zealand adults by Davern et al. [20]. On the contrary, Butler et al. [8] found a higher risk for anxiety/depression (but no risk for ADHD/disruptive behavior) for having access to one or three, but not zero or two child-specific places, in reference to access to four places, in US children and adolescents. While the former findings are in line with our results on the odds of emotional symptoms, their results for ADHD/disruptive behavior are difficult to compare. Unlike Butler et al. [8], we looked at hyperactivity/inattention and behavioral problems separately and observed an association of social infrastructure with hyperactivity but not with behavioral problems in the full sample.

Nevertheless, stratified analyses revealed differences in the association between SI and mental health by individual socioeconomic status (SES) and municipal socioeconomic deprivation (GISD). Low-SES children’s and adolescents’ mental health seemed to suffer more when lacking access to a park, which could not be found in medium- and high-SES children. This corresponds to other studies that stated that the mental health of low-SES children and adolescents from Lithuania, Scotland, England, and the USA benefits more from access to greenspace than mental health of higher-SES ones [22,23,24]. Results of this study underline this trend by yielding higher ORs for children and adolescents from high socioeconomically deprived areas when lacking access to a park or sports field (and to a lesser extent playgrounds) than for the other GISD strata. This common pattern by SES and GISD may be explained by the fact that children and adolescents with low SES are more likely to live in high socioeconomically deprived areas [11], to have no garden access [23], and to live further away from public greenspace [26]. Furthermore, those without garden access are more likely to live in a home environment with little total natural space and in high socioeconomically deprived areas [23] which are, in turn, less likely to have many public greenspaces [28] or natural spaces [23]. Interestingly, access to sports fields is associated with mental health in medium- and high-, but not low-SES children and adolescents and, simultaneously, in those living in high socioeconomically deprived but not medium or low deprived municipalities. Moreover, swimming pools and playgrounds are more strongly associated with mental health in high-, but not low- or medium-SES children and adolescents. The general low ORs in children and adolescents with medium SES and in those living in medium deprived municipalities may be due to the interplay between individual and area SES. A huge discrepancy between individual and area SES was shown to be detrimental to mental health [47]. In contrast, socioeconomically heterogeneous neighborhoods, which might be more likely to be found in medium deprived areas and around medium SES children and adolescents, have a beneficial influence [52]. This might explain the high ORs in high SES children and adolescents, as lacking access to a playground, sports field, and swimming pool is rare in this group and may lead more easily to a feeling of exclusion than in low-SES children and adolescents for whom it is more common to have no access to such places.

In addition to social inequality, some differences in the association between SI and mental health by urbanicity could be identified. That is to say, urban children and adolescents have higher odds of mental health difficulties when lacking park access compared to rural ones, which agrees with findings regarding residential greenness and behavioral problems in Belgian children [30]. A possible explanation may be that urban areas are more densely populated, are more built up, and have a higher road density [53], in addition to lacking natural elements, greenspaces, and tree cover outside of public parks [29]. Furthermore, this study revealed that the associations between child-specific places and mental health are higher in children compared to adolescents. This matches the findings of Vanaken and Danckaerts [19], who showed in their systematic review that a shorter distance is relevant for children, while the quality and the general proportion of greenspace in the area—which were not focus of this study—are more important for adolescents. Moreover, adolescents may favor other types of SI places such as shopping malls/streets [54] or cafés/restaurants [54]. In contrast to Richardson et al. [23], who observed some sex differences in the association between greenspaces and mental health in young children (aged four to six), our analysis indicated no interaction by sex. This might be due to the fact that Richardson et al. [23] differentiated between several types of greenspaces (total natural space, parks, and gardens), while we only looked at parks and included children and adolescents of a broader age range. It might be possible that sex differences are more pronounced in younger children than in adolescents. Future research should explore this more closely.

With regard to the single mental health domains, KiGGS SI (no access to playgrounds, sport fields, swimming pools, and parks) was mostly associated with higher total difficulties, emotional symptoms, hyperactivity/inattention, and peer problems within the full sample. With respect to the first three domains, this is consistent with previous systematic reviews [16,18,19].

The results of this study have to be interpreted in light of its limitations. First, a cross-sectional study was conducted which impedes causal inferences. Additionally, results of this study were not adjusted for multiple testing as it was an explorative study. Thus, confidence intervals and *p*-values have to be interpreted with caution. Since we decided to calculate unweighted regression estimates and descriptive statistics, prevalence cannot be considered representative of the population. Results of the SI score have to be interpreted with caution since the score was generated and applied within this study for the first time and has not been validated. Due to missing information, this study was also not able to examine the frequency of use or quality of SI or to adjust for parental mental health problems which could act as a confounder [8]. Parental mental health problems may be associated with children’s mental health problems and with selection into disadvantaged neighborhoods, which feature worse SI [5,6,10]. Thus, the effect estimates for the association between SI and children’s and adolescents’ mental health are likely to be smaller than in this study when additionally adjusting for parental mental health problems. Furthermore, we did not consider private greenspaces (e.g., gardens) to which 90% of the GerES V sample had access to. Despite the hierarchical data structure, a multilevel analysis could not be performed for the main analyses as it produced warning notifications due to too little variation on the municipal level. Nevertheless, the multilevel sensitivity analysis revealed similar ORs confirming the main results. Lastly, it has to be noted that the GerES V sample was smaller than the KiGGS wave 2 study, which may explain the large confidence intervals and high *p*-values. Moreover, the GerES V interview inquired about walking times in minutes to SI places, as opposed to a qualitative parental rating of reachability (yes vs. no) by self-administered questionnaires in KiGGS wave 2. This difference can also be assumed to reduce comparability.

Nevertheless, this study has several strengths worth noting. First, the KiGGS wave 2 and GerES V provide a large national representative sample using two-stage random selection from local population registries. Furthermore, the SDQ was validated in the KiGGS baseline study and has been commonly used in international research to describe children’s and adolescents’ mental health, enabling a comparison with other studies. Additionally, a comprehensive range of sensitivity analyses were conducted, which confirmed the results of the main analysis. This study focused not only on one level of social inequality but examined the role of both individual and municipal social inequality, adding important knowledge to existing literature. Lastly, this study makes important contributions to the existing state of evidence by examining SI, a hardly studied domain of built environment, and by investigating how SI affects children’s and adolescents’ mental health within different population groups.

## 5. Conclusions

In summary, lacking access to youth-specific SI is associated with mental health difficulties. Particularly children and adolescents living in high socioeconomically deprived municipalities and urban areas, as well as those with low SES, would benefit most from access to SI places, especially parks and sports fields. In addition, the mental health of high-SES children and adolescents is more strongly associated with the availability of playgrounds, swimming pools, and sports fields than the mental health of those with low SES. Therefore, sports fields, as well as parks and open natural spaces in general, should be foremost established in high socioeconomically deprived and urban areas, as well as near kindergartens and schools, to encourage their use by younger children. Furthermore, access should be facilitated by promoting the walkability and improving cycling lanes and public transport infrastructure in the areas surrounding youth-specific places.

There is still a need for further research on the influence of SI on children’s and adolescents’ mental health, especially with respect to areas other than greenspace and places that are more attractive to adolescents (such as shopping malls/streets, cafés, or restaurants) than the places examined in this study. Moreover, not only the availability but also the frequency of use, distance, and quality of SI places, as well as possible access barriers, should be examined in the future. Lastly, longitudinal studies are needed to explore mediators in the pathways from SI to mental health.

## Figures and Tables

**Table 1:** Sex-stratified characteristics of socio-demographic, social infrastructure, and mental health variables.

Socioeconomic Variable	Full Sample	Males	Females
***n* (%)**	12,624 (100)	6262 (49.60)	6362 (50.40)
**Age, mean ± SD**	10.14 ± 4.20	10.01 ± 4.16	10.28 ± 4.23
**Parental marital status, *n* (%)**			
Married, cohabitating	9707 (76.89)	4879 (77.91)	4828 (75.89)
Married, living separately	365 (2.89)	175 (2.79)	190 (2.99)
Not married	1487 (11.78)	715 (11.42)	772 (12.13)
Divorced	957 (7.58)	445 (7.11)	512 (8.05)
Widowed	108 (0.86)	48 (0.77)	60 (0.94)
**Migration background, *n* (%)**			
None	10,189 (80.71)	5104 (81.51)	5085 (79.93)
One-sided	1186 (9.39)	538 (8.59)	648 (10.19)
Two-sided	1249 (9.89)	620 (9.90)	629 (9.89)
**Socioeconomic Status (SES), *n* (%)**			
Low	1529 (12.11)	748 (11.95)	781 (12.28)
Medium	7803 (61.81)	3837(61.27)	3966 (62.34)
High	3292 (26.08)	1677 (26.78)	1615 (25.39)
**Socioeconomic deprivation of the municipality (GISD) ^1^, *n* (%)**			
Low deprivation	2341 (18.54)	1178 (18.81)	1163 (18.28)
Medium deprivation	7138 (56.54)	3531 (56.39)	3607 (56.70)
High deprivation	3145 (24.91)	1553 (24.80)	1592 (25.02)
**Living area ^1^, *n* (%)**			
Very central	4807 (38.08)	2351 (37.54)	2456 (38.60)
Central	3343 (26.48)	1688 (26.96)	1655 (26.01)
Peripheral	3647 (28.89)	1825 (29.14)	1822 (28.64)
Very peripheral	827 (6.55)	398 (6.36)	429 (6.74)
**Urbanicity ^1^, *n* (%)**			
Metropolitan city	2642 (20.93)	1288 (20.57)	1354 (21.28)
Medium city	3793 (30.05)	1833 (29.27)	1960 (30.81)
Larger small city	2420 (19.17)	1237 (19.75)	1183 (18.59)
Small city	2015 (15.96)	1011 (16.15)	1004 (15.78)
Rural area	1754 (13.89)	893 (14.26)	861 (13.53)
**Living in the municipality since birth, *n* (%)**			
Yes	8588 (68.03)	4253 (67.92)	4335 (68.14)
No	3800 (30.10)	1887 (30.13)	1913 (30.07)
Missing	236 (1.87)	122 (1.95)	114 (1.79)
**KiGGS SI Score, mean ± SD ^2^**	2.92 ± 1.11	2.93 ± 1.11	2.92 ± 1.12
**Single KiGGS SI variables** **Access to a …**			
**Playground, *n* (%)**			
No	1509 (11.95)	766 (12.23)	743 (11.68)
Yes	11,115 (88.05)	5496 (87.77)	5619 (88.32)
**Sports field, *n* (%)**			
No	2807 (22.24)	1275 (20.36)	1532 (24.08)
Yes	9817 (77.76)	4987 (79.64)	4830 (75.92)
**Swimming pool, *n* (%)**			
No	6410 (50.78)	3209 (51.25)	3201 (50.31)
Yes	6214 (49.22)	3053 (48.75)	3161 (49.69)
**Park, *n* (%)**			
No	2879 (22.81)	1469 (23.46)	1410 (22.16)
Yes	9745 (77.19)	4793 (76.54)	4952 (77.84)
**Mental health variables**			
**Total difficulties, *n* (%)**			
Normal	11,116 (88.05)	5413 (86.44)	5703 (89.64)
Borderline	728 (5.77)	409 (6.53)	319 (5.01)
Abnormal	780 (6.18)	440 (7.03)	340 (5.34)
**Emotional symptoms**			
Normal	10,784 (85.42)	5485 (87.59)	5299 (83.29)
Borderline	781 (6.19)	355 (5.67)	426 (6.70)
Abnormal	1059 (8.39)	422 (6.74)	637 (10.01)
**Behavioral problems**			
Normal	9449 (74.85)	4475 (71.46)	4974 (78.18)
Borderline	1707 (13.52)	938 (14.98)	769 (12.09)
Abnormal	1468 (11.63)	849 (13.56)	619 (9.73)
**Hyperactivity/Inattention**			
Normal	11,046 (87.50)	5272 (84.19)	5774 (90.76)
Borderline	639 (5.06)	378 (6.04)	261 (4.10)
Abnormal	939 (7.44)	612 (9.77)	327 (5.14)
**Peer problems**			
Normal	10,282 (81.45)	4976 (79.46)	5306 (83.40)
Borderline	1084 (8.59)	587 (9.37)	497 (7.81)
Abnormal	1258 (9.97)	699 (11.16)	559 (8.79)
**Prosocial behavior**			
Normal	11,552 (91.51)	5581 (89.12)	5971 (93.85)
Borderline	686 (5.43)	436 (6.96)	250 (3.93)
Abnormal	386 (3.06)	245 (3.91)	141 (2.22)

Abbreviations: *n*, number of observations; SD, standard deviation; CI, confidence interval. ^1^ Collected at municipal level. ^2^ The KiGGS SI score indicates the number of SI places (playground, sports field, swimming pool, and park) available for the child/adolescent.

**Table 2:** Multivariable analyses of the association between social infrastructure and children’s and adolescents’ mental health within the full sample.

SI Variable	Total Difficulties		Emotional Symptoms		Behavioral Problems		Hyperactivity/Inattention		Peer Problems		Prosocial Behavior	
*n* = 12,624	OR (95% CI)	*p*-Value	OR (95% CI)	*p*-Value	OR (95% CI)	*p*-Value	OR (95% CI)	*p*-Value	OR (95% CI)	*p*-Value	OR (95% CI)	*p*-Value
**KiGGS SI score ^1,2^**												
4	Ref.		Ref.		Ref.		Ref.		Ref.		Ref.	
3	1.14 (0.99, 1.33)	0.08	1.14 (0.98, 1.33)	0.08	1.05 (0.95, 1.17)	0.34	1.04 (0.90, 1.21)	0.58	1.01 (0.90, 1.13)	0.91	1.08 (0.94, 1.25)	0.29
2	1.37 (1.17, 1.61)	<0.001	1.22 (1.04, 1.43)	0.02	1.08 (0.95, 1.23)	0.23	1.26 (1.07, 1.48)	0.005	1.25 (1.08, 1.44)	0.003	1.12 (0.92, 1.35)	0.26
1	1.36 (1.13, 1.65)	0.002	1.24 (1.01, 1.51)	0.04	1.08 (0.92, 1.26)	0.38	1.18 (0.95, 1.46)	0.13	1.24 (1.03, 1.50)	0.02	1.21 (0.94, 1.55)	0.14
0	1.59 (1.19, 2.11)	0.002	1.22 (0.89, 1.68)	0.22	1.20 (0.97, 1.49)	0.10	1.40 (1.08, 1.81)	0.012	1.52 (1.17, 1.97)	0.002	1.33 (0.95, 1.85)	0.10
**Access to a …**	Ref. = Yes		Ref. = Yes		Ref. = Yes		Ref. = Yes		Ref. = Yes		Ref. = Yes	
**Playground ^2^**												
No	1.12 (0.95, 1.33)	0.19	1.03 (0.86, 1.23)	0.74	1.09 (0.96, 1.24)	0.17	1.06 (0.90, 1.25)	0.50	1.14 (0.98, 1.31)	0.09	1.15 (0.95, 1.39)	0.16
**Sports field ^2^**												
No	1.28 (1.12, 1.45)	<0.001	1.24 (1.08, 1.42)	0.003	1.01 (0.91, 1.12)	0.87	1.17 (1.03, 1.33)	0.02	1.37 (1.23, 1.52)	<0.001	1.13 (0.99, 1.30)	0.08
**Swimming pool ^2^**												
No	1.20 (1.06, 1.35)	0.004	1.12 (1.00, 1.26)	0.05	1.03 (0.95, 1.13)	0.45	1.10 (0.99, 1.22)	0.08	1.08 (0.97, 1.21)	0.15	1.09 (0.96, 1.25)	0.19
**Park ^2^**												
No	1.30 (1.15, 1.47)	< 0.001	1.08 (0.94, 1.24)	0.26	1.14 (1.03, 1.26)	0.01	1.27 (1.13, 1.43)	<0.001	1.18 (1.06, 1.31)	0.003	1.11 (0.97, 1.28)	0.14

Note: SI, social infrastructure; *n*, number of observations; OR, odds ratio; CI, confidence interval; Ref., reference category. ^1^ The KiGGS SI score indicates the number of SI places (playground, sports field, swimming pool, and park) available for the child/adolescent. ^2^ All estimates are from independent ordinal logistic regression models adjusted for individual socioeconomic status (SES), municipal social deprivation (German Index of Social Deprivation (GISD)), sex, age (in years), migration background, parental marital status, and spatial location.

**Table 3:** Multivariable analyses of the association between social infrastructure and total difficulties stratified by social inequality.

SI Variable	Low SES *n* = 1529		Medium SES *n* = 7803		High SES *n* = 3292		High Socioeconomic Deprivation ^1^ *n* = 3145		Medium Socioeconomic Deprivation ^1^ *n* = 7138		Low socioeconomic Deprivation ^1^ *n* = 2341	
	OR (95% CI)	*p*-Value	OR (95% CI)	*p*-Value	OR (95% CI)	*p*-Value	OR (95% CI)	*p*-Value	OR (95% CI)	*p*-Value	OR (95% CI)	*p*-Value
**KiGGS SI score ^2,3^**												
4	Ref.		Ref.		Ref.		Ref.		Ref.		Ref.	
3	1.21 (0.85, 1.73)	0.29	1.01 (0.85, 1.21)	0.88	1.65 (1.23, 2.23)	0.001	1.52 (1.08, 2.13)	0.02	1.06 (0.87, 1.28)	0.58	1.08 (0.76, 1.52)	0.66
2	1.73 (1.22, 2.44)	0.002	1.19 (0.96, 1.49)	0.12	1.81 (1.29, 2.55)	<0.001	1.75 (1.17, 2.62)	0.008	1.29 (1.06, 1.58)	0.01	1.24 (0.91, 1.70)	0.17
1	1.52 (1.00, 2.31)	0.05	1.24 (0.97, 1.58)	0.08	1.79 (1.00, 3.18)	0.05	1.71 (1.15, 2.55)	0.009	1.32 (1.03, 1.69)	0.03	1.06 (0.61, 1.87)	0.82
0	1.71 (0.95, 3.07)	0.08	1.45 (1.04, 2.02)	0.03	2.07 (1.01, 4.23)	0.047	2.86 (1.74, 4.70)	<0.001	1.08 (0.74, 1.59)	0.69	3.28 (1.36, 7.87)	0.01
**Access to a …**	Ref. = Yes		Ref. = Yes		Ref. = Yes		Ref. = Yes		Ref. = Yes		Ref. = Yes	
**Playground ^2^**												
No	1.08 (0.79, 1.49)	0.63	1.06 (0.87, 1.31)	0.55	1.49 (1.00, 2.20)	0.048	1.43 (1.07, 1.90)	0.02	0.97 (0.77, 1.23)	0.81	1.19 (0.74, 1.92)	0.46
**Sports field ^2^**												
No	1.29 (0.97, 1.70)	0.08	1.22 (1.04, 1.43)	0.01	1.55 (1.10, 2.17)	0.01	1.52 (1.19, 1.95)	0.001	1.18 (0.99, 1.40)	0.06	1.20 (0.86, 1.68)	0.27
**Swimming pool ^2^**												
No	1.11 (0.84, 1.46)	0.48	1.16 (1.00, 1.35)	0.054	1.51 (1.16, 1.97)	0.002	1.32 (1.01, 1.73)	0.04	1.13 (0.97, 1.32)	0.12	1.26 (0.92, 1.73)	0.14
**Park ^2^**												
No	1.83 (1.39, 2.40)	<0.001	1.18 (1.01, 1.39)	0.04	1.23 (0.93, 1.64)	0.15	1.62 (1.28, 2.06)	<0.001	1.21 (1.04, 1.42)	0.02	1.18 (0.90, 1.55)	0.22

Note: SI, social infrastructure; SES, socioeconomic status; *n*, number of observations; OR, odds ratio; CI, confidence interval; Ref., reference category. ^1^ Socioeconomic deprivation on municipal level. ^2^ All estimates are from independent ordinal logistic regression models adjusted for individual socioeconomic status (SES) (omitted in SES stratified analyses), municipal social deprivation (German Index of Social Deprivation (GISD)) (omitted in GISD stratified analyses), sex, age (in years), migration background, parental marital status, and spatial location. ^3^ The KiGGS SI score indicates the number of SI places (of playground, sports field, swimming pool, and park) available for the child/adolescent.

## Data Availability

Publicly available datasets were analyzed in this study. Data of the GISD can be found on https://data.gesis.org/sharing/#!Detail/10.7802/1460 (accessed on 24 June 2021). Access to the KiGGS and GerES V dataset can be provided upon request to interested researchers by the “Health Monitoring” Research Data Center at the Robert Koch-Institute (RKI) (https://www.rki.de/DE/Content/Forsch/FDZ/FDZ_node.html) (accessed on 24 June 2021) and by the German Environment Agency (Umweltbundesamt).

## Supplementary Materials

The following supporting information can be downloaded at https://www.mdpi.com/article/10.3390/ijerph19116760/s1: Table S1. Associations between the continuous social infrastructure variables and mental health in children and adolescents; Table S2. Multivariable analyses of the association between social infrastructure and mental health in children and adolescents with low socioeconomic status; Table S3. Multivariable analyses of the association between social infrastructure and mental health in children and adolescents with medium socioeconomic status; Table S4. Multivariable analyses of the association between social infrastructure and mental health in children and adolescents with high socioeconomic status; Table S5. Multivariable analyses of the association between social infrastructure and mental health in children and adolescents living in high socioeconomically deprived areas; Table S6. Multivariable analyses of the association between social infrastructure and mental health in children and adolescents living in medium socioeconomically deprived areas; Table S7. Multivariable analyses of the association between SI and mental health in children and adolescents living in low socioeconomically deprived areas; Table S8. Multivariable analyses of the association between social infrastructure and total difficulties, stratified by age; Table S9. Multivariable analyses of the association between social infrastructure and total difficulties in urban and rural children and adolescents; Table S10. Multivariable analyses of the association between social infrastructure and total difficulties, and results of sensitivity analyses of the full sample; Table S11. Demographic and socioeconomic characteristics of the GerES V sample; Table S12. Multivariable analyses of the association between social infrastructure and children’s and adolescents’ mental health within the GerES V sample.
