# Supplementary material for: Social Inequalities in the Association between Social Infrastructure and Mental Health: An Observational Cross-Sectional Analysis of Children and Adolescents in Germany
Source: Int J Environ Res Public Health. 2022 Jun 1;19(11):6760. doi: 10.3390/ijerph19116760 (PMC9180519; doi:10.3390/ijerph19116760)
Supplement: Supplementary file 1 [file ijerph-19-06760-s001.zip › ijerph-1701935-Supplementary.pdf]

Supplemental Material

Table S1. Associations between the continuous social infrastructure variables and mental health in children and adolescents

SI variable	Total difficulties		Emotional symptoms		Behavioral problems		Hyperactivity/ inattention		Peer problems		Prosocial behavior	
**Full sample n = 12624**	**OR (95% CI)**	**p-value**	**OR (95% CI)**	**p-value**	**OR (95% CI)**	**p-value**	**OR (95% CI)**	**p-value**	**OR (95% CI)**	**p-value**	**OR (95% CI)**	**p-value**
KiGGS SI score[1,2]	0.89 (0.84, 0.93)	<0.001	0.94 (0.89, 0.99)	0.02	0.97 (0.93, 1.01)	0.09	0.92 (0.88, 0.97)	<0.001	0.91 (0.87, 0.95)	<0.001	0.94 (0.89, 0.99)	0.02
**GerES V sample n = 2106**												
GerES V SI score[2,3]	0.88 (0.80, 0.97)	0.01	0.90 (0.82, 0.98)	0.02	0.97 (0.90, 1.04)	0.39	0.92 (0.83, 1.01)	0.09	0.98 (0.90, 1.07)	0.69	0.91 (0.81, 1.03)	0.13
**Distance to a...**												
Public playground[2]	1.05 (0.93, 1.17)	0.45	1.00 (0.89, 1.12)	0.94	0.99 (0.90, 1.09)	0.82	1.05 (0.94, 1.17)	0.42	0.96 (0.86, 1.07)	0.46	1.07 (0.93, 1.22)	0.35
Sports field[2]	1.10 (0.99, 1.21)	0.08	1.02 (0.93, 1.13)	0.65	1.03 (0.96, 1.12)	0.40	1.06 (0.96, 1.17)	0.27	1.04 (0.95, 1.14)	0.41	1.03 (0.91, 1.16)	0.67
Indoor playground[2]	1.21 (0.91, 1.61)	0.20	0.93 (0.75, 1.16)	0.53	1.25 (1.01, 1.56)	0.04	1.08 (0.82, 1.42)	0.61	1.05 (0.85, 1.30)	0.67	1.14 (0.82, 1.61)	0.44
Swimming pool[2]	1.08 (0.95, 1.22)	0.24	0.96 (0.86, 1.08)	0.49	1.03 (0.94, 1.14)	0.50	0.99 (0.88, 1.13)	0.93	1.02 (0.91, 1.13)	0.78	1.05 (0.90, 1.23)	0.51
Park/public greenspace[2]	1.08 (0.99, 1.18)	0.07	1.10 (1.02, 1.19)	0.01	1.03 (0.97, 1.10)	0.34	1.04 (0.96, 1.13)	0.37	1.04 (0.97, 1.13)	0.27	1.02 (0.91, 1.13)	0.75
Forest[2]	1.07 (0.98, 1.17)	0.16	1.13 (1.04, 1.23)	0.004	1.05 (0.98, 1.12)	0.17	1.03 (0.95, 1.13)	0.46	0.99 (0.92, 1.07)	0.82	1.15 (1.04, 1.28)	0.008
Blue space[2]	1.12 (1.03, 1.22)	0.007	1.12 (1.03, 1.21)	0.008	1.06 (0.99, 1.13)	0.10	1.03 (0.95, 1.13)	0.45	1.04 (0.97, 1.12)	0.27	1.05 (0.95, 1.16)	0.36
Public transport station[2]	1.14 (0.95, 1.38)	0.16	1.20 (1.01, 1.42)	0.04	1.02 (0.89, 1.18)	0.76	1.13 (0.94, 1.36)	0.18	1.10 (0.94, 1.29)	0.24	1.04 (0.84, 1.30)	0.72

Note: N number of observations, OR odds ratio, CI confidence interval, SI social infrastructure

[1]The KiGGS SI score indicates the number of SI places (of playground, sports field, swimming pool, park) available for the child/adolescent (higher value = more places available)

[2]All estimates are from independent ordinal logistic regression models adjusted for individual socioeconomic status (SES), municipal social deprivation (German Index of Social Deprivation (GISD)), sex, age (in years), migration background, parental marital status and spatial location

[3]the GerES V score indicates the number of SI places (of public playground, sportsground, indoor playground, swimming pool, public greenspace, forest, blue space and public transport station) reachable by foot within less than 10 minutes (higher value = more places available)

Table S2. Multivariable analyses of the association between social infrastructure and mental health in children and adolescents with low socioeconomic status

**SI variable**	**Total difficulties**		**Emotional symptoms**		**Behavioral problems**		**Hyperactivity/ inattention**		**Peer problems**		**Prosocial behavior**	
**n = 1529**	**OR (95% CI)**	**p-value**	**OR (95% CI)**	**p-value**	**OR (95% CI)**	**p-value**	**OR (95% CI)**	**p-value**	**OR (95% CI)**	**p-value**	**OR (95% CI)**	**p-value**
**KiGGS SI score[1,2]**												
4	Ref.		Ref.		Ref.		Ref.		Ref.		Ref.	
3	1.21 (0.85, 1.73)	0.29	1.29 (0.93, 1.80)	0.13	1.10 (0.82, 1.48)	0.54	1.18 (0.82, 1.71)	0.38	0.96 (0.75, 1.23)	0.74	1.20 (0.78, 1.83)	0.40
2	1.73 (1.22, 2.44)	0.002	1.65 (1.19, 2.30)	0.003	1.23 (0.88, 1.71)	0.23	1.71 (1.14, 2.57)	0.01	1.38 (1.02, 1.86)	0.04	1.52 (0.94, 2.47)	0.09
1	1.52 (1.00, 2.31)	0.05	1.79 (1.15, 2.77)	0.01	1.21 (0.80, 1.82)	0.36	1.22 (0.73, 2.03)	0.45	1.02 (0.68, 1.55)	0.91	1.60 (0.89, 2.87)	0.12
0	1.71 (0.95, 3.07)	0.08	1.59 (0.85, 2.98)	0.15	1.07 (0.66, 1.75)	0.78	1.52 (0.84, 2.74)	0.16	1.57 (0.90, 2.74)	0.11	2.36 (1.31, 4.25)	0.005
**Access to a …**												
	Ref. = Yes		Ref. = Yes		Ref. = Yes		Ref. = Yes		Ref. = Yes		Ref. = Yes	
**Playground[2]**												
No	1.08 (0.79, 1.49)	0.63	1.25 (0.86, 1.80)	0.24	1.14 (0.83, 1.57)	0.42	1.05 (0.73, 1.51)	0.79	1.06 (0.74, 1.52)	0.77	1.57 (1.06, 2.33)	0.03
**Sports field[2]**												
No	1.29 (0.97, 1.70)	0.08	1.26 (0.93, 1.70)	0.14	1.00 (0.79, 1.27)	0.99	1.21 (0.91, 1.60)	0.19	1.31 (1.04, 1.64)	0.02	1.33 (0.97, 1.82)	0.07
**Swimming pool[2]**												
No	1.11 (0.84, 1.46)	0.48	1.21 (0.92, 1.59)	0.17	0.91 (0.72, 1.15)	0.42	1.05 (0.78, 1.43)	0.74	1.03 (0.83, 1.27)	0.82	1.24 (0.87, 1.76)	0.23
**Park[2]**												
No	1.83 (1.39, 2.40)	<0.001	1.62 (1.23, 2.13)	<0.001	1.42 (1.11, 1.81)	0.006	1.64 (1.21, 2.21)	0.002	1.29 (1.00, 1.66)	0.05	1.59 (1.14, 2.21)	0.006

Note: SI social infrastructure, SES socioeconomic status, n number of observations, OR odds ratio, CI confidence interval, Ref. reference category

[1]the KiGGS SI score indicates the number of SI places (of playground, sports field, swimming pool, park) available for the child/adolescent

[2]All estimates are from independent ordinal logistic regression models adjusted for individual socioeconomic status (SES), municipal social deprivation (German Index of Social Deprivation (GISD)), sex, age (in years), migration background, parental marital status and spatial location

Table S3. Multivariable analyses of the association between social infrastructure and mental health in children and adolescents with medium socioeconomic status

SI variable	Total difficulties		Emotional symptoms		Behavioral problems		Hyperactivity/ inattention		Peer problems		Prosocial behavior	
**n = 7803**	**OR (95% CI)**	**p-value**	**OR (95% CI)**	**p-value**	**OR (95% CI)**	**p-value**	**OR (95% CI)**	**p-value**	**OR (95% CI)**	**p-value**	**OR (95% CI)**	**p-value**
**KiGGS SI score[12]**												
4	Ref.		Ref.		Ref.		Ref.		Ref.		Ref.	
3	1.01 (0.85, 1.21)	0.88	1.04 (0.86, 1.26)	0.67	0.97 (0.85, 1.11)	0.69	1.01 (0.84, 1.22)	0.90	1.00 (0.85,1.17)	0.95	1.03 (0.83, 1.27)	0.80
2	1.19 (0.96, 1.49)	0.12	1.08 (0.86, 1.36)	0.49	0.97 (0.82, 1.14)	0.70	1.16 (0.95, 1.41)	0.14	1.16 (0.97, 1.40)	0.11	1.08 (0.85, 1.38)	0.53
1	1.24 (0.97, 1.58)	0.08	1.11 (0.87, 1.42)	0.38	0.95 (0.78, 1.16)	0.63	1.12 (0.87, 1.44)	0.37	1.28 (1.02, 1.60)	0.03	1.13 (0.83, 1.54)	0.45
0	1.45 (1.04, 2.02)	0.03	1.19 (0.83, 1.71)	0.35	1.27 (1.00, 1.62)	0.048	1.47 (1.08, 2.00)	0.02	1.51 (1.08, 2.11)	0.02	1.17 (0.76, 1.80)	0.47
**Access to a …**												
	Ref. = Yes		Ref. = Yes		Ref. = Yes		Ref. = Yes		Ref. = Yes		Ref. = Yes	
**Playground[2]**												
No	1.06 (0.87, 1.31)	0.55	0.95 (0.77, 1.17)	0.62	1.08 (0.93, 1.25)	0.33	1.08 (0.88, 1.32)	0.48	1.09 (0.90, 1.31)	0.38	1.03 (0.81, 1.33)	0.80
**Sports field[2]**												
No	1.22 (1.04, 1.43)	0.01	1.22 (1.03, 1.45)	0.02	0.98 (0.86, 1.11)	0.75	1.06 (0.92, 1.24)	0.42	1.41 (1.21, 1.64)	<0.001	1.13 (0.95, 1.35)	0.16
**Swimming pool[2]**												
No	1.16 (1.00, 1.35)	0.054	1.08 (0.93, 1.25)	0.31	1.00 (0.89, 1.11)	0.94	1.12 (0.99, 1.27)	0.07	1.09 (0.95, 1.25)	0.21	1.05 (0.88, 1.26)	0.57
**Park[2]**												
No	1.18 (1.01, 1.39)	0.04	1.01 (0.85, 1.20)	0.93	1.09 (0.97, 1.23)	0.16	1.27 (1.09, 1.48)	0.002	1.14 (1.00, 1.30)	0.056	1.07 (0.91, 1.26)	0.42

Note: SI social infrastructure, SES socioeconomic status, n number of observations, OR odds ratio, CI confidence interval, Ref. reference category

[1]the KiGGS SI score indicates the number of SI places (of playground, sports field, swimming pool, park) available for the child/adolescent

[2]All estimates are from independent ordinal logistic regression models adjusted for individual socioeconomic status (SES), municipal social deprivation (German Index of Social Deprivation (GISD)), sex, age (in years), migration background, parental marital status and spatial location

Table S4. Multivariable analyses of the association between social infrastructure and mental health in children and adolescents with high socioeconomic status

**SI variable**	**Total difficulties**		**Emotional symptoms**		**Behavioral problems**		**Hyperactivity/ inattention**		**Peer problems**		**Prosocial behavior**	
**n = 3292**	**OR (95% CI)**	**p-value**	**OR (95% CI)**	**p-value**	**OR (95% CI)**	**p-value**	**OR (95% CI)**	**p-value**	**OR (95% CI)**	**p-value**	**OR (95% CI)**	**p-value**
**KiGGS SI score**[1,2]												
4	Ref.		Ref.		Ref.		Ref.		Ref.		Ref.	
3	1.65 (1.23, 2.23)	0.001	1.44 (1.10, 1.90)	0.009	1.24 (1.03, 1.51)	0.03	1.06 (0.80, 1.39)	0.70	1.07 (0.84, 1.36)	0.58	1.17 (0.91, 1.51)	0.23
2	1.81 (1.29, 2.55)	<0.001	1.45 (1.04, 2.02)	0.03	1.41 (1.12, 1.79)	0.004	1.31 (0.92, 1.86)	0.134	1.47 (1.12, 1.93)	0.006	1.00 (0.62, 1.60)	0.98
1	1.79 (1.00, 3.18)	0.05	1.23 (0.73, 2.05)	0.43	1.46 (1.00, 2.14)	0.05	1.53 (0.91, 2.57)	0.11	1.42 (0.92, 2.19)	0.12	1.27 (0.72, 2.24)	0.41
0	2.07 (1.01, 4.23)	0.047	0.95 (0.44, 2.08)	0.90	0.97 (0.53, 1.78)	0.91	0.94 (0.41, 2.17)	0.88	1.52 (0.76, 3.03)	0.24	0.97 (0.42, 2.26)	0.94
**Access to a …**												
	Ref. = Yes		Ref. = Yes		Ref. = Yes		Ref. = Yes		Ref. = Yes		Ref. = Yes	
**Playground**[2]												
No	1.49 (1.00, 2.20)	0.048	1.18 (0.84, 1.67)	0.34	1.13 (0.85, 1.52)	0.41	1.03 (0.72, 1.46)	0.89	1.48 (1.08, 2.05)	0.02	1.18 (0.75, 1.85)	0.47
**Sports field**[2]												
No	1.55 (1.10, 2.17)	0.01	1.29 (0.97, 1.71)	0.08	1.23 (0.89, 1.43)	0.32	1.63 (1.18, 2.24)	0.003	1.30 (1.00, 1.69)	0.052	0.98 (0.64, 1.48)	0.91
**Swimming pool**[2]												
No	1.51 (1.16, 1.97)	0.002	1.23 (0.98, 1.55)	0.08	1.28 (1.08, 1.50)	0.004	1.10 (0.88, 1.38)	0.40	1.15 (0.93, 1.41)	0.20	1.14 (0.89, 1.46)	0.29
**Park**[2]												
No	1.23 (0.93, 1.64)	0.15	0.94 (0.69, 1.28)	0.70	1.13 (0.91, 1.41)	0.28	0.96 (0.70, 1.33)	0.82	1.23 (0.93, 1.63)	0.14	0.93 (0.65, 1.33)	0.67

Note: SI social infrastructure, SES socioeconomic status, n number of observations, OR odds ratio, CI confidence interval, Ref. reference category

[1]the KiGGS SI score indicates the number of SI places (of playground, sports field, swimming pool, park) available for the child/adolescent

[2]All estimates are from independent ordinal logistic regression models adjusted for individual socioeconomic status (SES), municipal social deprivation (German Index of Social Deprivation (GISD)), sex, age (in years), migration background, parental marital status and spatial location

Table S5. Multivariable analyses of the association between social infrastructure and mental health in children and adolescents living in high socioeconomically deprived areas

**SI variable**	**Total difficulties**		**Emotional symptoms**		**Behavioral problems**		**Hyperactivity/ inattention**		**Peer problems**		**Prosocial behavior**	
**n = 3145**	**OR (95% CI)**	**p-value**	**OR (95% CI)**	**p-value**	**OR (95% CI)**	**p-value**	**OR (95% CI)**	**p-value**	**OR (95% CI)**	**p-value**	**OR (95% CI)**	**p-value**
**KiGGS SI score[12]**												
4	Ref.		Ref.		Ref.		Ref.		Ref.		Ref.	
3	1.52 (1.08, 2.13)	0.02	1.47 (1.03, 2.08)	0.03	1.09 (0.88, 1.35)	0.44	1.25 (0.94, 1.65)	0.12	1.03 (0.78, 1.36)	0.82	1.17 (0.82, 1.69)	0.38
2	1.75 (1.17, 2.62)	0.008	1.49 (1.03, 2.15)	0.04	1.21 (0.93, 1.56)	0.15	1.58 (1.10, 2.27)	0.01	1.41 (1.05, 1.90)	0.02	1.70 (1.15, 2.53)	0.01
1	1.71 (1.15, 2.55)	0.009	1.53 (1.01, 2.32)	0.047	1.03 (0.73, 1.46)	0.85	1.34 (0.86: 2.10)	0.20	1.29 (0.90, 1.86)	0.17	1.28 (0.74, 2.21)	0.37
0	2.86 (1.74, 4.70)	<0.001	1.65 (0.94, 2.91)	0.08	1.77 (1.16, 2.69)	0.009	2.19 (1.35, 3.57)	0.002	2.00 (1.22, 3.28)	0.007	2.51 (1.40, 4.53)	0.003
**Access to a …**												
	Ref. = Yes		Ref. = Yes		Ref. = Yes		Ref. = Yes		Ref. = Yes		Ref. = Yes	
**Playground[2]**												
No	1.43 (1.07, 1.90)	0.02	1.18 (0.84, 1.64)	0.34	1.23 (0.98, 1.55)	0.08	1.50 (1.17, 1.92)	0.002	1.27 (0.94, 1.72)	0.11	1.47 (1.02, 2.12	0.04
**Sports field[2]**												
No	1.52 (1.19, 1.95)	0.001	1.45 (1.14, 1.84)	0.003	1.06 (0.85, 1.31)	0.61	1.25 (0.96, 1.62)	0.09	1.46 (1.19, 1.80)	<0.001	1.35 (1.02, 1.77)	0.03
**Swimming pool[2]**												
No	1.32 (1.01, 1.73)	0.04	1.16 (0.89,1.50)	0.26	1.09 (0.91, 1.31)	0.34	1.21 (0.94, 1.55)	0.13	1.15 (0.91, 1.45)	0.23	1.41 (1.00, 1.97)	0.048
**Park[2]**												
No	1.62 (1.28, 2.06)	<0.001	1.21 (0.93, 1.57)	0.16	1.31 (1.04, 1.65)	0.02	1.48 (1.15, 1.91)	0.003	1.33 (1.06, 1.67)	0.02	1.41 (1.08, 1.82)	0.01

Note: SI social infrastructure, n number of observations, OR odds ratio, CI confidence interval, Ref. reference category

[1]the KiGGS SI score indicates the number of SI places (of playground, sports field, swimming pool, park) available for the child/adolescent

[2]All estimates are from independent ordinal logistic regression models adjusted for individual socioeconomic status (SES), municipal social deprivation (German Index of Social Deprivation (GISD)), sex, age (in years), migration background, parental marital status and spatial location

Table S6. Multivariable analyses of the association between social infrastructure and mental health in children and adolescents living in medium socioeconomically deprived areas

SI variable	Total difficulties		Emotional symptoms		Behavioral problems		Hyperactivity/ inattention		Peer problems		Prosocial behavior	
**n = 7138**	**OR (95% CI)**	**p-value**	**OR (95% CI)**	**p-value**	**OR (95% CI)**	**p-value**	**OR (95% CI)**	**p-value**	**OR (95% CI)**	**p-value**	**OR (95% CI)**	**p-value**
**KiGGS SI score[12]**												
4	Ref.		Ref.		Ref.		Ref.		Ref.		Ref.	
3	1.06 (0.87, 1.28)	0.58	1.08 (0.89, 1.31)	0.45	0.98 (0.86, 1.12)	0.75	0.95 (0.77, 1.16)	0.59	1.03 (0.89, 1.19)	0.68	1.18 (0.98, 1.43)	0.08
2	1.29 (1.06, 1.58)	0.01	1.17 (0.95, 1.44)	0.14	1.01 (0.85, 1.20)	0.93	1.23 (1.00, 1.51)	0.056	1.25 (1.02, 1.52)	0.03	1.09 (0.87, 1.38)	0.45
1	1.32 (1.03, 1.69)	0.03	1.12 (0.87, 1.46)	0.38	1.04 (0.85, 1.26)	0.71	1.11 (0.84, 1.48)	0.46	1.26 (0.98, 1.63)	0.07	1.39 (1.02, 1.89)	0.04
0	1.08 (0.74, 1.59)	0.69	1.02 (0.66, 1.56)	0.94	0.96 (0.73, 1.26)	0.76	1.11 (0.81, 1.52)	0.53	1.27 (0.91, 1.78)	0.17	0.83 (0.52, 1.30)	0.41
**Access to a …**												
	Ref. = Yes		Ref. = Yes		Ref. = Yes		Ref. = Yes		Ref. = Yes		Ref. = Yes	
**Playground[2]**												
No	0.97 (0.77, 1.23)	0.81	0.95 (0.76, 1.18)	0.64	1.02 (0.86, 1.22)	0.78	0.92 (0.73, 1.15)	0.46	1.00 (0.83, 1.20)	0.96	0.98 (0.76, 1.26)	0.88
**Sports field[2]**												
No	1.18 (0.99, 1.40)	0.06	1.14 (0.93, 1.38)	0.21	0.95 (0.83, 1.10)	0.50	1.14 (0.98, 1.33)	0.09	1.40 (1.21, 1.63)	<0.001	1.15 (0.97, 1.37)	0.12
**Swimming pool[2]**												
No	1.13 (0.97, 1.32)	0.12	1.08 (0.93, 1.26)	0.32	0.99 (0.88, 1.11)	0.81	1.05 (0.91, 1.20)	0.53	1.06 (0.92, 1.22)	0.44	1.04 (0.89, 1.23)	0.60
**Park[2]**												
No	1.21 (1.04, 1.42)	0.02	1.04 (0.87, 1.24)	0.67	1.07 (0.94, 1.21)	0.33	1.24 (1.06, 1.45)	0.008	1.16 (1.01, 1.33)	0.04	1.05 (0.88, 1.25)	0.59

Note: SI social infrastructure, n number of observations, OR odds ratio, CI confidence interval, Ref. reference category

[1]the KiGGS SI score indicates the number of SI places (of playground, sports field, swimming pool, park) available for the child/adolescent

[2]All estimates are from independent ordinal logistic regression models adjusted for individual socioeconomic status (SES), municipal social deprivation (German Index of Social Deprivation (GISD)), sex, age (in years), migration background, parental marital status and spatial location

Table S7. Multivariable analyses of the association between SI and mental health in children and adolescents living in low socioeconomically deprived areas

**SI variable**	**Total difficulties**		**Emotional symptoms**		**Behavioral problems**		**Hyperactivity/ inattention**		**Peer problems**		**Prosocial behavior**	
**n = 2341**	**OR (95% CI)**	**p-value**	**OR (95% CI)**	**p-value**	**OR (95% CI)**	**p-value**	**OR (95% CI)**	**p-value**	**OR (95% CI)**	**p-value**	**OR (95% CI)**	**p-value**
**KiGGS SI score**[12]												
4	Ref.		Ref.		Ref.		Ref.		Ref.		Ref.	
3	1.08 (0.76, 1.52)	0.66	1.05 (0.76, 1.44)	0.77	1.26 (0.96, 1.66)	0.10	1.17 (0.83, 1.65)	0.37	0.93 (0.71, 1.22)	0.61	0.78 (0.60, 1.01)	0.18
2	1.24 (0.91, 1.70)	0.17	1.14 (0.79, 1.64)	0.47	1.14 (0.87, 1.49)	0.33	1.01 (0.72, 1.44)	0.93	1.07 (0.80, 1.43)	0.63	0.67 (0.37, 1.22)	0.19
1	1.06 (0.61, 1.87)	0.82	1.30 (0.82, 2.07)	0.25	1.39 (0.83, 2.32)	0.20	1.34 (0.82, 2.18)	0.23	1.09 (0.67, 1.80)	0.72	0.63 (0.31, 1.28)	0.02
0	3.28 (1.36, 7.87)	0.01	2.37 (0.98, 5.76)	0.056	1.36 (0.73, 2.55)	0.32	1.65 (0.61, 4.45)	0.31	2.09 (0.87, 5.04)	0.10	2.76 (1.17, 6.51)	0.44
**Access to a …**												
	Ref. = Yes		Ref. = Yes		Ref. = Yes		Ref. = Yes		Ref. = Yes		Ref. = Yes	
**Playground**[2]												
No	1.19 (0.74, 1.92)	0.46	1.21 (0.71, 2.05)	0.48	1.13 (0.80, 1.60)	0.49	0.74 (0.42, 1.32)	0.30	1.51 (1.08, 2.10)	0.02	1.32 (0.81, 2.17)	0.26
**Sports field**[2]												
No	1.20 (0.86, 1.68)	0.27	1.32 (0.98, 1.78)	0.07	1.16 (0.99, 1.37)	0.07	1.15 (0.73, 1.79)	0.54	1.08 (0.88, 1.33)	0.43	0.75 (0.49, 1.12)	0.15
**Swimming pool**[2]												
No	1.26 (0.92, 1.73)	0.14	1.20 (0.94, 1.53)	0.13	1.13 (0.91, 1.40)	0.26	1.23 (0.96, 1.57)	0.10	1.08 (0.84, 1.37)	0.55	0.90 (0.68, 1.19)	0.44
**Park**[2]												
No	1.18 (0.90, 1.55)	0.22	1.08 (0.75, 1.55)	0.67	1.17 (0.92, 1.47)	0.19	1.13 (0.83, 1.54)	0.43	1.03 (0.81, 1.31)	0.83	0.96 (0.62, 1.49)	0.85

Note: SI social infrastructure, n number of observations, OR odds ratio, CI confidence interval, Ref. reference category, n.o. no observations

[1]the KiGGS SI score indicates the number of SI places (of playground, sports field, swimming pool, park) available for the child/adolescent

[2]All estimates are from independent ordinal logistic regression models adjusted for individual socioeconomic status (SES), municipal social deprivation (German Index of Social Deprivation (GISD)), sex, age (in years), migration background, parental marital status and spatial location

Table S8. Multivariable analyses of the association between social infrastructure and total difficulties, stratified by age

SI variable	Children aged <11 years n = 6419		Adolescents aged ≥11 years n = 6205		Adolescents aged ≥11 years (adjusted for SSS instead of individual SES) n = 5703	
**n = 6419**	**OR (95% CI)**	**p-value**	**OR (95% CI)**	**p-value**	**OR (95% CI)**	**p-value**
**KiGGS SI score[12]**						
4	Ref.		Ref.		Ref.	
3	1.41 (1.13, 1.76)	0.003	0.95 (0.78, 1.15)	0.57	0.96 (0.79, 1.17)	0.67
2	1.55 (1.24, 1.95)	<0.001	1.26 (1.01, 1.57)	0.04	1.30 (1.03, 1.63)	0.03
1	1.85 (1.42, 2.42)	<0.001	0.94 (0.71, 1.25)	0.68	0.86 (0.63, 1.18)	0.36
0	2.09 (1.45, 3.01)	<0.001	1.20 (0.76, 1.91)	0.43	1.29 (0.80, 2.10)	0.30
**Access to a …**						
	Ref. = Yes		Ref. = Yes		Ref. = Yes	
**Playground[2]**						
No	1.22 (0.96, 1.54)	0.11	1.07 (0.86, 1.34)	0.56	1.11 (0.89, 1.38)	0.38
**Sports field[2]**						
No	1.39 (1.16, 1.67)	<0.001	1.15 (0.94, 1.40)	0.17	1.09 (0.89, 1.34)	0.40
**Swimming pool[2]**						
No	1.47 (1.24, 1.74)	<0.001	0.97 (0.83, 1.13)	0.66	0.97 (0.83, 1.14)	0.72
**Park[2]**						
No	1.41 (1.18, 1.68)	<0.001	1.17 (0.96, 1.41)	0.11	1.20 (0.97, 1.48)	0.09

Note: SI social infrastructure, N number of observations, SSS subjective socioeconomic status, OR odds ratio, CI confidence interval, Ref. reference category
[1]the KiGGS SI score indicates the number of SI places (of playground, sportsground, swimming pool, park) available for the child/adolescent
[2]All estimates are from independent ordinal logistic regression models adjusted for individual socioeconomic status (SES) (or SSS in case of the second adolescents' subgroup), municipal social deprivation (German Index of Social Deprivation (GISD)), sex, age, migration background, parental marital status and spatial location

Table S9. Multivariable analyses of the association between social infrastructure and total difficulties in urban and rural children and adolescents

**SI variable**	**Urban children and adolescents n = 6435**		**Rural children and adolescents n = 6189**	
	**OR (95% CI)**	**p-value**	**OR (95% CI)**	**p-value**
**KiGGS SI score**[12]				
4	Ref.		Ref.	
3	1.20 (0.99, 1.46)	0.06	1.11 (0.88, 1.38)	0.38
2	1.37 (1.09, 1.72)	0.007	1.39 (1.10, 1.74)	0.006
1	1.58 (1.19, 2.10)	0.002	1.25 (0.95, 1.64)	0.10
0	1.96 (1.24, 3.10)	0.004	1.46 (1.03, 2.08)	0.04
**Access to a …**				
	Ref. = Yes		Ref. = Yes	
**Playground**[2]				
No	1.23 (0.97, 1.56)	0.09	1.06 (0.84, 1.35)	0.61
**Sports field**[2]				
No	1.31 (1.07, 1.61)	0.01	1.27 (1.08, 1.49)	0.004
**Swimming pool**[2]				
No	1.30 (1.10, 1.53)	0.002	1.15 (0.96, 1.37)	0.12
**Park**[2]				
No	1.43 (1.19, 1.73)	<0.001	1.23 (1.05, 1.44)	0.01

Note: SI social infrastructure, N number of observations, OR odds ratio, CI confidence interval, Ref. reference category,
[1]the KiGGS SI score indicates the number of SI places (of playground, sportsground, swimming pool, park) available for the child/adolescent
[2]All estimates are from independent ordinal logistic regression models adjusted for individual socioeconomic status (SES), municipal social deprivation (German Index of Social Deprivation (GISD)), sex, age (in years), migration background, parental marital status and spatial location

Table S10. Multivariable analyses of the association between social infrastructure and total difficulties, results of sensitivity analyses of the full sample

SI variable	Additionally adjusted for 'living in the municipality since birth'[1]		Adjusted for the single SES indicators[2]		Effect estimates obtained by multilevel analysis[3]	
**n = 12388**	**OR (95% CI)**	**p-value**	**OR (95% CI)**	**p-value**	**OR (95% CI)**	**p-value**
**KiGGS SI score[4]**						
4	Ref.		Ref.		Ref.	
3	1.13 (0.97, 1.31)	0.12	1.13 (0.97, 1.31)	0.12	1.13 (0.97, 1.31)	0.11
2	1.37 (1.16, 1.60)	<0.001	1.35 (1.15, 1.58)	<0.001	1.39 (1.17, 1.64)	<0.001
1	1.34 (1.11, 1.63)	0.003	1.33 (1.10, 1.60)	0.004	1.46 (1.18, 1.81)	<0.001
0	1.61 (1.21, 2.14)	0.001	1.55 (1.17, 2.06)	0.003	1.55 (1.17, 2.06)	0.002
**Single KiGGS SI variables**						
**Access to a …**	Ref. = Yes		Ref. = Yes		Ref. = Yes	
**Playground**						
No	1.08 (0.91, 1.28)	0.35	1.11 (0.94, 1.32)	0.21	1.10 (0.92, 1.30)	0.30
**Sports field**						
No	1.27 (1.12, 1.45)	<0.001	1.27 (1.11, 1.44)	<0.001	1.29 (1.12, 1.47)	<0.001
**Swimming pool**						
No	1.20 (1.06, 1.36)	0.003	1.18 (1.05, 1.34)	0.008	1.23 (1.09, 1.39)	0.001
**Park**						
No	1.32 (1.17, 1.49)	<0.001	1.28 (1.13, 1.44)	<0.001	1.35 (1.18, 1.54)	<0.001

Note: SI social infrastructure, N number of observations, OR odds ratio, CI confidence interval, Ref. reference category

[1]All estimates are from independent ordinal logistic regression models adjusted for individual socioeconomic status (SES), municipal social deprivation (German Index of Social Deprivation (GISD)), sex, age (in years), migration background, parental marital status, spatial location and living in the municipality since birth

[2]All estimates are from independent ordinal logistic regression models adjusted for the SES indicators (occupational status, education and income), municipal social deprivation (German Index of Social Deprivation (GISD)), sex, age (in years), migration background, parental marital status and spatial location

[3]All estimates are from independent multilevel ordinal logistic regression models adjusted for individual socioeconomic status (SES), municipal social deprivation (German Index of Social Deprivation (GISD)), sex, age (in years), migration background, parental marital status and spatial location

[4]the KiGGS SI score indicates the number of SI places (of playground, sportsground, swimming pool, park) available for the child/adolescent

Table S11. Demographic and socioeconomic characteristics of the GerES V sample

Socioeconomic and demographic characteristics	Full sample
**Full sample, n (%)**	2106 (100)
**Sex, n (%)**	
Male	1043 (49.53)
Female	1063 (50.47)
**Age, mean ±SD**	10.11 ±4.14
**Parental marital Status, n (%)**	
Married, cohabitating	1654 (78.54)
married, living separately	52 (2.47)
not married	233 (11.06)
divorced	144 (6.84)
widowed	23 (1.09)
**Migration background, n (%)**	
None	1691 (80.29)
One-sided	203 (9.64)
Two-sided	212 (10.07)
**Socioeconomic Status (SES), n (%)**	
Low	238 (11.30)
Medium	1286 (61.06)
High	582 (27.64)
**Social deprivation of the municipality (GISD)[1], n (%)**	
Lowly deprived	390 (18.52)
Medium deprived	1179 (55.98)
Highly deprived	537 (25.50)
**Spatial location[1], n (%)**	
Very central	787 (37.37)
Central	590 (28.02)

Peripheral	577 (27.40)
Very peripheral	152 (7.22)
**Urbanicity[1], n (%)**	
Metropolitan city (≥100,000 inhabitants)	440 (20.89)
Medium city (20,000 – <100,000 inhabitants)	628 (29.82)
Larger small city (10,000 – <20,000 inhabitants)	369 (17.52)
Small city (5,000 – <10,000 inhabitants)	285 (13.53)
Rural area (<5,000 inhabitants)	384 (18.23)
**Mental health**	
**Total difficulties, n (%)**	
Normal	1822 (86.51)
Borderline/Abnormal	284 (13.49)
**Emotional symptoms, n (%)**	
Normal	1775 (84.28)
Borderline/Abnormal	331 (15.72)
**Behavioral symptoms, n (%)**	
Normal	1557 (73.93)
Borderline/Abnormal	549 (26.07)
**Hyperactivity/Inattention, n (%)**	
Normal	1818 (86.32)
Borderline/Abnormal	288 (13.68)
**Peer problems, n (%)**	
Normal	1700 (80.72)
Borderline/Abnormal	406 (19.28)
**Prosocial behavior, n (%)**	
Normal	1929 (91.60)
Borderline/Abnormal	177 (8.40)
**GerES V SI score[2], mean ±SD**	4.04 ±1.37

N number of observations, SD standard deviation, CI confidence interval, SI social infrastructure
[1]Collected at the municipality level
[2]the GerES V score indicates the number of SI places (of public playground, sportsground, indoor playground, swimming pool, public greenspace, forest, blue space and public transport station) reachable by foot within less than 10 minutes

Table S12. Multivariable analyses of the association between social infrastructure and children's and adolescents' mental health within the GerES V sample

SI variable	Total difficulties		Emotional symptoms		Behavioral problems		Hyperactivity/ inattention		Peer problems		Prosocial behavior	
**N = 2106**	**OR (95% CI)**	**p-value**	**OR (95% CI)**	**p-value**	**OR (95% CI)**	**p-value**	**OR (95% CI)**	**p-value**	**OR (95% CI)**	**p-value**	**OR (95% CI)**	**p-value**
**GerEs V SI score[12]**												
6	Ref.		Ref.		Ref.		Ref.		Ref.		Ref.	
5	0.80 (0.51, 1.25)	0.32	0.99 (0.66, 1.49)	0.97	0.81 (0.59, 1.12)	0.20	0.92 (0.59, 1.42)	0.70	0.94 (0.66, 1.35)	0.75	0.87 (0.51, 1.47)	0.59
4	1.18 (0.77, 1.80)	0.46	1.22 (0.82, 1.82)	0.33	0.97 (0.70, 1.33)	0.83	0.99 (0.64, 1.51)	0.95	0.96 (0.67, 1.37)	0.81	1.06 (0.63, 1.76)	0.83
3	1.20 (0.76, 1.89)	0.45	1.27 (0.82, 1.97)	0.28	0.94 (0.66, 1.33)	0.72	0.98 (0.61, 1.55)	0.92	1.09 (0.74, 1.60)	0.67	1.24 (0.72, 2.14)	0.44
2	1.44 (0.87, 2.39)	0.16	1.48 (0.92, 2.39)	0.11	1.10 (0.75, 1.63)	0.62	1.30 (0.79, 2.14)	0.30	0.99 (0.63, 1.56)	0.98	1.40 (0.77, 2.56)	0.27
1	1.68 (0.86, 3.28)	0.13	1.69 (0.90, 3.19)	0.11	1.01 (0.59, 1.75)	0.97	1.73 (0.89, 3.34)	0.11	1.04 (0.55, 1.96)	0.91	1.18 (0.48, 2.89)	0.72
**Public playground[2]**												
1 – 5 min.	Ref.		Ref.		Ref.		Ref.		Ref.		Ref.	
6 – 10 min.	1.43 (1.04, 1.96)	0.03	1.03 (0.76, 1.40)	0.84	1.23 (0.96, 1.57)	0.10	1.10 (0.79, 1.51)	0.58	1.06 (0.80, 1.40)	0.70	1.29 (0.87, 1.91)	0.20
11 – 20 min.	1.15 (0.76, 1.74)	0.51	1.18 (0.80, 1.74)	0.41	0.96 (0.70, 1.33)	0.82	1.09 (0.73, 1.62)	0.68	1.05 (0.73, 1.51)	0.78	1.46 (0.91, 2.32)	0.12
21 – 30 min.	0.29 (0.07, 1.17)	0.08	0.51 (0.18, 1.47)	0.22	0.38 (0.16, 0.93)	0.04	1.38 (0.65, 2.91)	0.40	1.09 (0.52, 2.29)	0.82	0.52 (0.12, 2.22)	0.38
More than 30 min.	1.35 (0.77, 2.36)	0.29	0.97 (0.54, 1.76)	0.93	1.13 (0.71, 1.80)	0.60	1.13 (0.64, 2.00)	0.66	0.66 (0.36, 1.21)	0.18	1.18 (0.57, 2.44)	0.65
**Sports field[2]**												
1 – 5 min.	Ref.		Ref.		Ref.		Ref.		Ref.		Ref.	
6 – 10 min.	0.96 (0.67, 1.36)	0.81	1.17 (0.85, 1.61)	0.33	1.08 (0.82, 1.41)	0.59	1.02 (0.71, 1.45)	0.93	0.97 (0.72, 1.30)	0.81	1.43 (0.93, 2.18)	0.10
11 – 20 min.	1.03 (0.71, 1.48)	0.90	0.98 (0.70, 1.38)	0.91	1.20 (0.91, 1.59)	0.20	1.02 (0.71, 1.47)	0.91	0.92 (0.67, 1.26)	0.58	1.32 (0.84, 2.08)	0.23
21 – 30 min.	1.31 (0.78, 2.19)	0.31	0.84 (0.48, 1.47)	0.54	0.97 (0.63, 1.48)	0.88	1.36 (0.82, 2.25)	0.24	1.20 (0.76, 1.89)	0.43	0.97 (0.47, 2.03)	0.94
More than 30 min.	1.42 (0.93, 2.17)	0.11	1.28 (0.85, 1.94)	0.24	1.16 (0.82, 1.66)	0.40	1.21 (0.78, 1.87)	0.39	1.18 (0.80, 1.74)	0.42	1.23 (0.69, 2.19)	0.48

**Indoor playground**[2]												
1 – 10 min.	Ref.		Ref.		Ref.		Ref.		Ref.		Ref.	
11 – 20 min.	0.33	0.16	0.62	0.34	0.98	0.98	0.23	0.051	1.13	0.82	0.98	0.98
	(0.07, 1.52)		(0.23: 1.68)		(0.31, 3.15)		(0.05, 1.00)		(0.41, 3.15)		(0.17, 5.73)	
21 – 30 min.	1.25	0.72	0.30	0.04	2.07	0.20	0.95	0.93	0.65	0.43	1.20	0.84
	(0.36, 4.35)		(0.10, 0.92)		(0.68, 6.28)		(0.30, 2.99)		(0.23, 1.88)		(0.21, 6.74)	
More than 30 min.	1.10	0.86	0.57	0.17	1.92	0.19	0.71	0.48	1.08	0.86	1.37	0.67
	(0.39, 3.12)		(0.26, 1.28)		(0.73, 5.05)		(0.27, 1.84)		(0.47, 2.48)		(0.32, 5.84)	
**Swimming pool**[2]												
1 – 10 min.	Ref.		Ref.		Ref.		Ref.		Ref.		Ref.	
11 – 20 min.	1.01	0.98	0.93	0.73	0.82	0.32	1.00	0.99	0.69	0.09	1.07	0.83
	(0.61, 1.67)		(0.60, 1.42)		(0.57, 1.20)		(0.62, 1.62)		(0.45, 1.05)		(0.59, 1.94)	
21 – 30 min.	0.95	0.84	0.65	0.08	0.82	0.33	0.60	0.07	0.80	0.29	0.84	0.61
	(0.56, 1.62)		(0.41, 1.04)		(0.56, 1.22)		(0.35, 1.04)		(0.52, 1.21)		(0.44, 1.63)	
More than 30 min.	1.21	0.38	0.87	0.45	1.00	0.99	0.97	0.87	0.90	0.55	1.16	0.56
	(0.80, 1.83)		(0.61, 1.25)		(0.74, 1.37)		(0.64, 1.45)		(0.64, 1.26)		(0.70, 1.93)	
**Park/public greenspace**[2]												
1 – 5 min.	Ref.		Ref.		Ref.		Ref.		Ref.		Ref.	
6 – 10 min.	0.81	0.30	1.07	0.73	1.02	0.92	0.87	0.47	0.86	0.34	0.86	0.53
	(0.55, 1.20)		(0.75, 1.51)		(0.77, 1.34)		(0.59, 1.28)		(0.62, 1.18)		(0.55, 1.37)	
11 – 20 min.	1.07	0.75	1.68	0.004	0.94	0.71	1.12	0.58	0.77	0.16	0.88	0.60
	(0.71, 1.60)		(1.18, 2.40)		(0.68, 1.30)		(0.75, 1.67)		(0.53, 1.11)		(0.53, 1.45)	
21 – 30 min.	1.15	0.65	1.22	0.47	1.02	0.94	1.40	0.24	1.50	0.12	0.82	0.62
	(0.64, 2.05)		(0.71, 2.11)		(0.64, 1.63)		(0.80, 2.45)		(0.91, 2.47)		(0.36, 1.83)	
More than 30 min.	1.37	0.08	1.41	0.045	1.18	0.25	1.09	0.63	1.17	0.31	1.13	0.59
	(0.97, 1.93)		(1.01, 1.98)		(0.89, 1.56)		(0.77, 1.55)		(0.86, 1.59)		(0.73, 1.74)	
**Forest**[2]												
1 – 5 min.	Ref.		Ref.		Ref.		Ref.		Ref.		Ref.	
6 – 10 min.	0.78	0.23	0.92	0.68	0.99	0.94	0.95	0.79	0.81	0.21	2.38	<0.001
	(0.53, 1.16)		(0.63, 1.35)		(0.74, 1.33)		(0.65, 1.39)		(0.58, 1.13)		(1.48, 3.85)	
11 – 20 min.	0.84	0.367	1.06	0.76	1.04	0.77	1.07	0.74	0.83	0.27	2.06	0.004
	(0.57, 1.23)		(0.73, 1.53)		(0.78, 1.40)		(0.73, 1.55)		(0.60, 1.15)		(1.26, 3.36)	
21 – 30 min.	1.07	0.77	1.46	0.09	1.01	0.96	1.30	0.26	0.75	0.19	1.47	0.26
	(0.67, 1.72)		(0.95, 2.25)		(0.70, 1.46)		(0.82, 2.06)		(0.49, 1.15)		(0.76, 2.87)	
More than 30 min.	1.25	0.23	1.54	0.02	1.24	0.15	1.07	0.74	0.99	0.94	2.29	0.001
	(0.87, 1.79)		(1.09, 2.17)		(0.93, 1.65)		(0.73, 1.55)		(0.72, 1.36)		(1.40, 3.76)	

**Blue space**[2]												
1 – 5 min.	Ref.		Ref.		Ref.		Ref.		Ref.		Ref.	
6 – 10 min.	1.11 (0.75, 1.64)	0.62	1.18 (0.82, 1.69)	0.37	1.07 (0.80, 1.43)	0.67	0.75 (0.51, 1.11)	0.15	1.11 (0.80, 1.54)	0.54	0.84 (0.52, 1.36)	0.49
11 – 20 min.	1.61 (1.09, 2.38)	0.02	0.96 (0.65, 1.42)	0.83	1.14 (0.84, 1.55)	0.40	1.21 (0.83, 1.75)	0.32	1.28 (0.91, 1.79)	0.16	1.12 (0.70, 1.80)	0.64
21 – 30 min.	1.20 (0.71, 2.02)	0.50	1.49 (0.95, 2.34)	0.09	0.84 (0.55, 1.27)	0.40	0.87 (0.52, 1.46)	0.59	1.18 (0.76, 1.83)	0.47	1.41 (0.79, 2.51)	0.24
More than 30 min.	1.60 (1.12, 2.30)	0.01	1.56 (1.12, 2.17)	0.009	1.33 (1.01, 1.75)	0.045	1.09 (0.76, 1.55)	0.65	1.18 (0.86, 1.62)	0.31	1.10 (0.71, 1.71)	0.66
**Public transport station**[2]												
1 – 5 min.	Ref.		Ref.		Ref.		Ref.		Ref.		Ref.	
6 – 10 min.	1.03 (0.75, 1.41)	0.85	1.22 (0.91, 1.62)	0.18	1.12 (0.89, 1.42)	0.34	0.93 (0.68, 1.28)	0.65	1.32 (1.02, 1.72)	0.04	1.17 (0.81, 1.70)	0.41
11 – 20 min.	1.22 (0.75, 1.99)	0.42	1.55 (1.00, 2.39)	0.049	0.89 (0.60, 1.31)	0.55	1.35 (0.85, 2.14)	0.20	0.99 (0.64, 1.56)	0.98	1.02 (0.56, 1.86)	0.94
More than 20 min.	2.23 (0.95, 5.23)	0.07	1.29 (0.48, 3.52)	0.62	1.26 (0.58, 2.77)	0.56	1.97 (0.84, 4.64)	0.12	1.25 (0.49, 3.18)	0.63	0.88 (0.21, 3.72)	0.86

Note: N number of observations, OR odds ratio, CI confidence interval, SI social infrastructure, Ref. reference category, min. minutes

[1]the GerES V score indicates the number of SI places (of public playground, sportsground, indoor playground, swimming pool, public greenspace, forest, blue space and public transport station) reachable by foot within less than 10 minutes

[2]All estimates are from independent ordinal logistic regression models adjusted for individual socioeconomic status (SES), municipal social deprivation (German Index of Social Deprivation (GISD)), sex, age (in years), migration background, parental marital status and spatial location
